# Acoustic Sensing Based on Online Handwritten Signature Verification †

**DOI:** 10.3390/s22239343

**Published:** 2022-11-30

**Authors:** Mengqi Chen, Jiawei Lin, Yongpan Zou, Kaishun Wu

**Affiliations:** College of Computer Science and Software Engineering, Shenzhen University, Yuehai Street, Shenzhen 518061, China

**Keywords:** acoustic sensor, acoustic sensing, signature verification

## Abstract

Handwritten signatures are widely used for identity authorization. However, verifying handwritten signatures is cumbersome in practice due to the dependency on extra drawing tools such as a digitizer, and because the false acceptance of a forged signature can cause damage to property. Therefore, exploring a way to balance the security and user experiment of handwritten signatures is critical. In this paper, we propose a handheld signature verification scheme called SilentSign, which leverages acoustic sensors (i.e., microphone and speaker) in mobile devices. Compared to the previous online signature verification system, it provides handy and safe paper-based signature verification services. The prime notion is to utilize the acoustic signals that are bounced back via a pen tip to depict a user’s signing pattern. We designed the signal modulation stratagem carefully to guarantee high performance, developed a distance measurement algorithm based on phase shift, and trained a verification model. In comparison with the traditional signature verification scheme, SilentSign allows users to sign more conveniently as well as invisibly. To evaluate SilentSign in various settings, we conducted comprehensive experiments with 35 participants. Our results reveal that SilentSign can attain 98.2% AUC and 1.25% EER. We note that a shorter conference version of this paper was presented in Percom (2019). Our initial conference paper did not finish the complete experiment. This manuscript has been revised and provided additional experiments to the conference proceedings; for example, by including System Robustness, Computational Overhead, etc.

## 1. Introduction

Handwritten signature verification (HSV for short) aims to verify whether a given signature is real or fake [1]. It possesses considerable significance in our daily life, such as when handling banking business and signing important documents. Handwritten signatures, however, are reported to be faked in plenty of critical fraud cases due to the existing defects in HSV techniques. The latest JP Morgan report reveals that paper check fraud has reached the top ranking among various payment procedures in recent years [2]. This fuels the growth of HSV research. According to the signature collection approach, HSV schemes can be classified as online verification and offline verification [3]. Offline verification denotes signing the person’s name on paper materials and verifying the signature afterward. Such an HSV happens in instances where paper material documents need to be signed, such as cheques and contracts. However, this lacks a real-time record of the signing procedure and makes the signature easily counterfeited. On the other hand, the development of hardware illuminates some novel HSV technologies for researchers, enabling mobile devices to perform online verification of signers and strengthen the protection of HSV. The fundamental rule is to capture the dynamic characteristics of the signing motion, such as tip pressure, order of strokes, and signing speed. It is possible to consider these characteristics as one’s identity, as they represent unique patterns that are specific to each individual. The result is that in online HSV schemes, instead of just verifying the signature shape, the process of signing itself can be used to verify the identity of signers, thus strengthening the integrity of the handwritten signature verification process.

Within the scope of online HSV, a drawing input device, such as a digitizer or touchpad, is used in most previous schemes [3,4,5,6], in which users can sign their signatures on an electromagnetic resonance technology [7] plane with a specialized stylus. The limitations of such schemes are that they require specialized hardware and are therefore unsuitable when users must sign on paper-like materials. In other words, real-time verification services cannot be provided in offline schemes. Online may be preferred with respect to offline verification. Vision-based methods record the signing process for verification. However, it raises security concerns because attackers can easily mimic a person’s signature due to the leaked recording. Prior research [8] seized the opportunity of the sensing capability in popular wearable devices, using a a smartwatch that captures the motion of the wrist via HSV IMU sensors. Compared to other research [3,4,5,6], this novel approach solves hardware inaccessibility deficiencies. However, it also has the following weaknesses. Owing to computing resource restrictions, HSV systems in smartwatches have to offload recorded data to another powerful computing device, such as a mobile or a tablet. In other words, an HSV system needs to be installed on two separate devices. Even with the extra computing capacity, the smartwatch also needs another device that runs applications such as online banking services as a result of its restricted screen size. Moreover, this method requires the signer to wear a device, which may level the user experience.

Accordingly, we raise the following question: *can we design an HSV scheme that can be signed on paper material with only an off-the-shelf tool without the user wearing or touching any additional hardware?* SilentSign is proposed by the above insight in this paper. It can be applied to any portable device with acoustic sensors and transformed into an online HSV system. Compared to vision-based technology, acoustic sensing has several advantages, such as privacy insensitivity, lower storage, and computational overhead. SilentSign takes advantage of the internal speaker-microphone pair prevalent on commercial portable devices without making hardware-level modifications or equipping any additional device.

A designed ultrasonic sequence will be transmitted to the air. The vertical distance change of a signature pen tip can be extracted from the phase shift of the reflecting echo. Then, similarity features are extracted between the reference and questioned signature. These features are utilized to train a classifier to verify. When a new recording of an unknown signature is presented, our trained classification model will compare it with the enrolled signatures, determining whether the unknown signature is genuine or forged. The contributions of our work can be summarized as follows:

- We propose an acoustic-based HSV approach that can be easily implemented on popular mobile devices. It replaces customized HSV devices with handy hardware and enables the online HSV service for the requirement of paper materials signing. Compared to previous work, a signer does not need to wear additional equipment;
- We design a machine learning model for HSV by integrating imaging similarity features that describe different sign-trajectories motion patterns. Evaluations show that our method achieves favorable performance while the model enrolls a new user without retraining;
- Finally, we conduct extensive experiments to evaluate the performance of our system in different settings. The results show that SilentSign can distinguish genuine and forged signatures with an AUC of 98.2% and an EER of 2.37%.

We note that a shorter conference version [9] of this article was presented in Percom (2019). The conference version is the first step of research to explore the feasibility of using acoustic sensing in handwritten signature verification. Beyond that scope, this manuscript also considers a more comprehensive assessment of SilentSign. Compared to the conference version, we have extended the evaluation section with assessments of smartphone orientation, noise influence, robustness against attacks, and computational overhead. We also make a comparison with the state of the art in comparison with existing works. The results show that SilentSign outperforms SOTA in acoustic sensing ways.

The remainder of this paper is organized as follows. We outline the related work in Section 2. We provide the background information and overview the required architecture in Section 3. Section 4 and Section 5 introduce the techniques used in system design and in the construction of the verification models. We evaluate the performance of the system in Section 6. In Section 7 and Section 8, we discuss the remaining problems and future work, and conclude this paper, respectively.

## 2. Related Works

### 2.1. Handwritten Signature Verification

Based on the signature collection approach, existing HSV techniques can be classified into two categories: online and offline [10,11]. The offline system uses offline acquisition devices such as a scanner or camera to obtain static images as input data. The verification process is performed after the writing process. Compared to the offline approaches, the online approaches are preferred due to their security. Generally, online approaches are based on dynamic characteristics such as azimuth, tip pressure, and altitude. Numerous digitizing devices (e.g., tablets, smartwatches, and IMU-equipped pens) have been used to capture stylus or wrist motion data in previous research [3,4,8,12,13,14]. Recently, device-free approaches have drawn the attention of researchers. AirSign [15] uses motion and acoustic sensors to sign signatures in the air using smartphones without the need for special hardware. However, this work uses only a finger as the signing tool, making it inappropriate to sign on paper with a pen. ASSV [16] takes advantage of acoustic sensors to track the trajectory of a moving pen tip. The tracking direction and the authentication model are the main differences between ASSV and SilentSign. SilentSign makes use of a well-designed transmit sequence to measure the track of a pen’s tip. As a result, it integrates both benefits of online and offline systems, such as dynamic data and device-free signing on the paper, and performs fine-grained accuracy.

### 2.2. Biometric Authentication on Smart Devices

Biometric behavior has been used on mobile and wearable devices to authenticate the legal user. Not only familiar static biometrics (fingerprint, iris, face, etc.), but also numerous novel biometrics, such as keystrokes, and breath, have been proven to be feasible to apply in human authentication [17,18,19]. Other features such as dental [20,21] and face [22,23], heart rate [18,24], and breath [19] have also been used in authentication on mobile or wearable devices. In SmileAuth [21] and Bilock [20], authors leverage dental collision sound as a unique feature for authentication. EchoFace [23] and EchoPrint [22] combine acoustic sensing and image recognition to allow even older devices to use secure face recognition. Heart rate [18,24] and breath [19,25] are characteristics that attackers have difficulty in imitating. However, biometric behavior may be preferred with respect to authentication due to unique habits caused by living circumstances. VibWrite [17] tries to capture the dynamic movement of the hand when a particular motion is performed by a user toward the tablet screen to authenticate the user’s legitimacy. AirAuth [26] utilizes a short-range depth camera to capture gestures in the air for authentication. Handwritten signatures as a biometric behavior have been used for a long time to confirm a legitimate individual. In addition, it has irreplaceable applications for special occasions.

### 2.3. Acoustic Human–Computer Interaction

Acoustic sensing as a non-contact means of human–computer interaction has broad application scenarios. Due to the spread of the range and the smaller amount of processed data, sound-based sensing is more advantageous in motion detection or localization, such as gestures, by using mobile and wearable devices [27,28,29,30,31,32,33,34], and indoor localization [35,36]. FingerIO [28] uses an inaudible OFDM-modulated sound frame to locate the finger movement by detecting the change of two consecutive frames. VSkin [32] leverages the structure-borne and air-borne sound paths to detect gestures performed on the surface of the smartphone. MilliSonic [37] leverages FMCW (frequency-modulated continuous wave) and a novel localization algorithm to implement fine-grained 3D tracking for VR headsets. BeepBeep [35] measures the distance between devices by acoustic range. In Ref. [36], the authors use a chirp-based range sonar to achieve the location. EarphoneTrack [38] can transform earphones into hand-tracking controllers.

In this paper, SilentSign combines the phase-based and frame-based approach by using the Zadoff–Chu code, which has good auto-correlation properties and the ability to track phase changes. It also has the advantages of a high refresh rate and directly correlating with the movement of a pen tip.

## 3. System Architecture

### 3.1. Design Goal

Signatures, as a form of identification in authorizing a cheque or document or concluding a letter, play an important role in our daily life. Existing approaches leveraging specialized hardware (e.g., digitizers or cameras) are either unsafe or unsuitable for pen-paper scenarios. Therefore, our design goal is to build a convenient and highly accurate signature verification system that is available on existing smartphones without requiring any external device. Previous works either leverage digitizers or cameras. To achieve this goal, we implement a highly accurate acoustic sensing system to capture the movement of the pen tip while signing the signature. Moreover, combined with a machine learning algorithm, we utilize a training approach that can accurately verify newly enrolled signatures without requiring any re-training operation. The SilentSign system has proper trade-offs between security and convenience.

### 3.2. Overview

**Figure 1:**
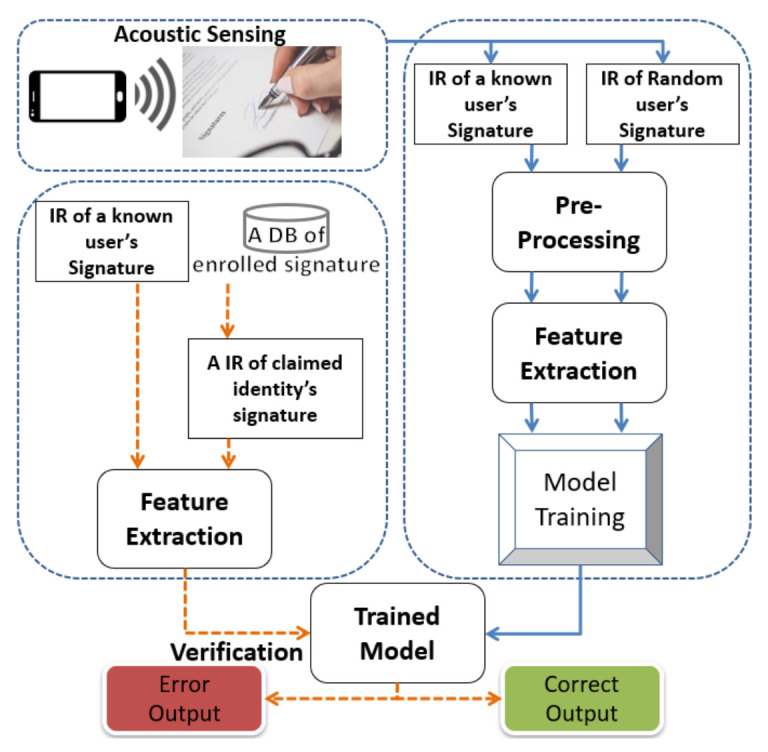
The system architecture of SilentSign.

Figure 1 We demonstrate the system architecture of SilentSign in Figure 1. First, the built-in speaker and microphone on the mobile are turned on simultaneously before signing. A sequence of designed ultrasonic sounds will transmit to the air, reflect on the surrounding objects, and arrive at the microphone. We extract the phase shift and impulse response from the echo. These metrics indicate the change in distance of the tip of the pen in the vertical direction. It should be noted that distance variation can be considered as a grayscale figure because distance variation is a 2-D vector. This clarifies that we can feed an authentication classifier with image similarity between forged and genuine features. The above general idea paves the way for designing SilentSign. There are two usage phases in our system: one is signature enrollment, and the other is verification. In signature enrollment, an adequate number of reference signatures, which are supplied by newcomers, will be stored in the system as template samples. In signature verification, the system runs a verification procedure to tackle a signature that is signed by the questioned individual. In both phases, SilentSign will continually calculate the phase shift and the response of the echo while the user is signing. Finally, the authentication result comes from comparing the questioned signature and the stored reference signature.

## 4. System Design

### 4.1. Acoustic Sensing

We list the following considerations before designing the sensing component:

(1) Commercial digitizers and touch screens in the current market can reach a sampling rate of 75∼200 Hz and track a pen tip with at least 5 mm resolution. Our acoustic sensing component should be close to achieving the above verification accuracy;
(2) The echoes received by the microphone are a multipath signal convoluted with surrounding objects. Thus, differentiating the path with respect to the moving pen from others is a challenge;
(3) For a better user experience, the transmitted sound should be inaudible.

**Figure 2:**
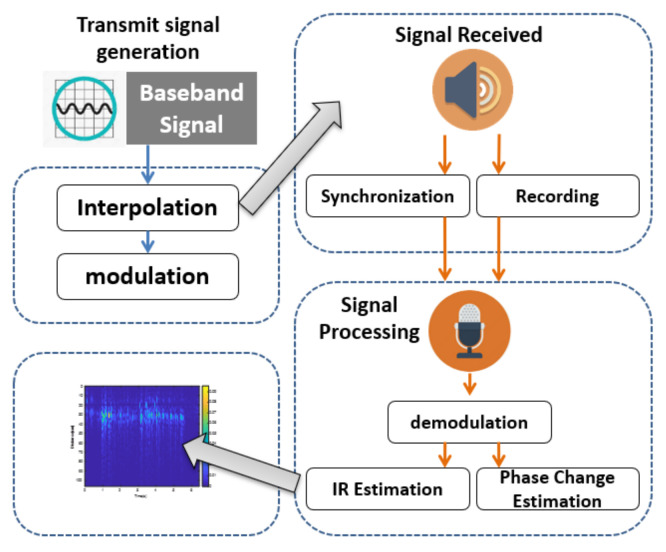
The overview of a sample acoustic sensing component.

As shown in Figure 2, we designed an acoustic sensing scheme to meet the above requirements. There are three main components, namely signal generation, signal reception, and distance measurement. The signal generation component is responsible for creating inaudible modulated ultrasound signals. Reflected sound signals from surrounding objects are received by the signal reception component through the microphone, and then the sender and receiver are synchronized. The distance measurement component demodulates the received signals to extract the distance variations between the moving tip of the pen and the smartphone. This component includes three steps to estimate different impulse responses, estimate the phase shift, and format the impulse response. We describe each component in detail as follows.

### 4.2. Transmit Signal Generation

The phase-coded (PC) sequence is normally used to decrease radio frequency interference in radar applications. A PC waveform (*S*) can be separated into *N* bit segments {s[1],...,s[N]}; we call each segment [i] a chip. It is difficult to design an appropriate PC sequence with perfect resolution due to the innumerable choices of the phase of each chip. Designing a sequence with an outstanding autocorrelation property is a feasible way. Therefore, in our work, we choose 127 Zadoff–Chu bits, which are a complex-valued mathematical sequence. In prior work [32] it has been shown that the ZC sequence 127 bits can track moving objects with an average movement distance error of 3.59 mm and 3 kHz, which is quite approximate to the digitizer property. We processed the raw 127 bits ZC sequence and transformed it into 17∼23 kHz. Subsequently, the frequency domain interpolation was achieved by inserting zeros into the sequence $ZC_{127bits}$ until its length reached 1024 bits. The resulting sequence had a bandwidth of 6 kHz and the corresponding sampling rate was 48 kHz. We further fit such a sequence into the passband, which was realized by using real bits and artificially inserted bits to multiply with a carrier. The carrier frequency was 20.25 kHz. Eventually, the transmit signal $S_{ZCT}$ was tuned in the frequency range of 17.29∼23.25 kHz.

### 4.3. Adaptive Energy-Based LOS Detection

Traditional sonar systems can synchronize the sending and recording operations of the signal. After starting the recording operation, the sonar concurrently manages the buffering of the received signal and calculates the distance of the reflected path. Synchronization of the sender and receiver provides a reference for the delay between the sending and receiving time of the initial pulse through line-of-sight (LOS). Without synchronization, the delay between the initial pulse and the first received pulse may not accurately present the time interval of pulse travel through LOS, which will cause a deviation from the subsequent distance measurement. Due to the compatibility issue of the Android operating system, it is difficult to synchronize the speaker and microphone.

To solve this problem, we add 24,000 points of zero at the very beginning of $S_{ZCT}$, leaving a time of half a second for the receiver to start the recording operation. This allows the microphone to receive the first pulse completely. Since our acoustic sensing system is based on monostatic sonar, the speaker and microphone are fixed on the smartphone, which means we already know the length between the speaker and the microphone. In other words, we know the length of the LOS and the travel time of the first pulse. After we found the LOS of the first pulse, we used it as the start-time reference by simply adding a fixed delay. The fixed delay is based on the distance between the speaker and the microphone.

**Figure 3:**
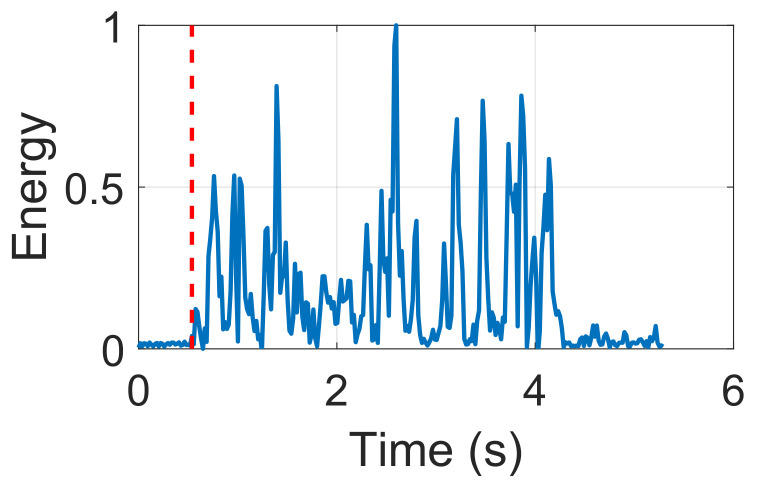
Adaptive energy-based initial pulse detection.

We used an adaptive energy-based LOS detection approach to identify the first impulse. Then, we also inserted $ZC_{1024bits}$ after the 24,000 zeros. The $ZC_{1024bits}$ is a good quality autocorrelation. As soon as we began documenting, the correlation function, i.e., $IRF\left(t\right)=ZC_{R}^{*}\left(-t\right)\times ZC_{1024bits}\left(t\right)$, was used to find the first impulse. Specifically, $ZC_{R}^{*}\left(-t\right)$ was the conjugation of the baseband signal. Figure 3 shows the first impulse detected by autocorrelation, which has a low level of the side lobe. The LOS path is the first spike in Figure 3.

Subsequently, we should pinpoint the exact spot of the maximum LOS point. Thus, we went through the LOS path from the very beginning and leveraged the energy-based approach to find the maximum point. Specifically, we borrowed the idea of an adaptive energy-based event detection scheme, which was done in prior work [20], to deal with a wide range of noise levels. Limited by page space, we omit detailed equations and refer readers to previous work [20]. According to Figure 3, the red line is a rough starting point, and the peak is within the next 1024 points on the LOS path. Ultimately, we applied a maximum function to discover the exact position of this peak. The LOS path is a baseline for the following distance measurement. By adding a fixed delay to the position of the LOS path, we used this position as the starting point for the impulse response.

### 4.4. Distance Measurement

#### 4.4.1. Differential IR Estimations

An acoustic sender typically transmits a known sequence in modern sonar systems. Sound signals propagate through the air to meet objects within the detection range and are reflected to the receiver in a very short time interval. In this process, the acoustics are reflected from different paths, resulting in varying time delays. That is, the received signals are a mixture of different components. After synchronization between the receiver and the sender, we demodulate the received signals in the baseband (denoted $S_{ZCR}\left(t\right)$) with a low-pass filter. We then use the cross-correlation function to separate different paths and compute the impulse response (IR) as follows.

(1) $$IRF\left(t\right)=S_{ZCR}^{*}\left(-t\right)*S_{ZCT}\left(t\right)$$

Each peak in IRF(t) represents a propagation component with a corresponding delay. Thus, these changes are achieved by subtracting adjacent IRs as follows.

(2) $$\Delta IR=IR_{t∼t+W-1}-IR_{t+W∼t+2W-1}$$

Moreover, we propose a standard energy threshold algorithm to detect movements of a pen tip to reduce the computational overhead. Specifically, the position of a maximum point in ΔIR represents the distance between a pen tip and the smartphone. However, the frame refresh rate is about 46.875 Hz since the window size is 1024, which is less than that of a digitizer (i.e., 75∼200 Hz). To increase this parameter, we incorporate the estimation of the phase shift as follows.

#### 4.4.2. Estimation of the Phase Shift

By detecting moving paths, we calculate the path coefficient to improve the refresh rate. Ultimately, the distance variation of a moving pen tip can be determined by incorporating the phase shift of the path coefficient. The path coefficient describes changes in the amplitude and phase of a given path, which can be calculated as follows:

(3) $$\begin{array}{c} h_{t}\left[n_{i}\right]=\sum_{l=0}^{N_{ZC-1}}S_{ZCR}\left[t+l\right]*S_{ZCT}^{*}\left[\left(l-n_{i}\right)\;\;\mathrm{mod}\;N_{ZC}\right] \end{array}$$

where $N_{ZC}$ represents the length of the ZC sequence equal to 1024; $n_{i}$ represents the position of the maximum point in ΔIR. To save the computation cost, we only calculate the coefficient of the moving path. Differential IR estimations indicate the path related to the moving tip of the pen. Taking into account the phase shift of this path, the distance variation can be determined as follows.

(4) $$\begin{array}{c} d_{i}\left(t\right)-d_{i}\left(0\right)=-\frac{\sum_{i=1}^{t}\Delta \theta_{i-1}^{i}}{2\pi }*\lambda_{c} \end{array}$$

where $\lambda_{c}$ is the sound wavelength $\lambda_{c}=c/f_{c}$ and

(5) $$\begin{array}{c} \Delta \theta_{t-1}^{t}=\frac{Q_{h_{t}}}{I_{h_{t}}}-\frac{Q_{h_{t-1}}}{I_{h_{t-1}}} \end{array}$$

**Figure 4:**
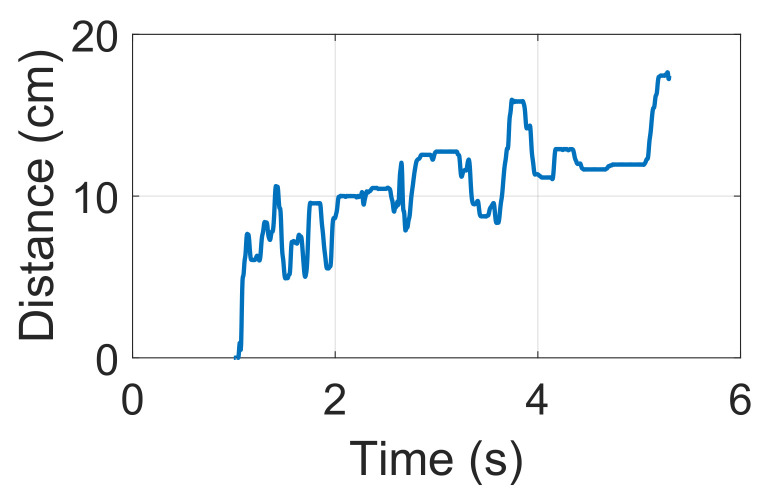
Distance variation during the signing.

We combine low sampling rate IR response estimation with distance change. Then, by determining the maximum point in the IR estimation, the distance variation is a two-dimensional array, as shown in Figure 4.

#### 4.4.3. Format IR Estimations

As the initial position is uncertain, We perform normalization to the value of the IR estimation. Then, we make use of the IR Format Algorithm 1 to eliminate the beginning points not related to the detection of the movement and detect the starting point as the center of the array. $IR_{formated}$ is the training sample for the following discussion.
**Algorithm 1:** IR Format  **Input**: $IR_{norm}$  **Output**: $IR_{formated}$1c=0;2r=0;3$cLen=len(IR_{norm}\left[:\right]\left[0\right]);$4**while** $sum(IR_{norm}\left[:\right]\left[c\right])==0$**do**5  | c++6**end**7$\left[result,r\right]=max(IR_{norm}\left[:\right]\left[c\right]);$8$IR_{formated}=\left[zeros\left(cLen-r\right);IR_{norm}\left[1:r+cLen,:\right]\right]$

## 5. Authentication Model

### 5.1. Feature Extraction

Traditional similarity features such as structural similarity (SSIM), peak signal-to-noise ratio (PSNR), mean squared error (MSE), and Hausdorff distance have been widely used to measure image similarity [39,40]. Therefore, in our system, we calculated the above four features and generated a vector of characteristic similarity of four dimensions (S={SSIM,PSNR,MSE,HAUSDORFF}) from two formatted and scaled IR responses $IR_{A}$, $IR_{B}$. Based on whether these two types of IR are generated from the same genuine signature dataset, we labeled the feature vector as *genuine* or *forged*.

### 5.2. Model Training

**Figure 5:**
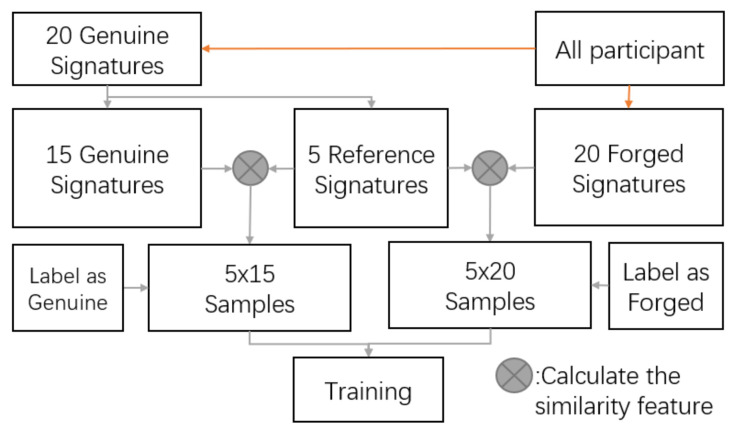
Model training design.

During the enrollment phase of the training dataset, the user provides a reasonable number of signatures to calculate the feature vector *F*. Each Genuine is paired with another Genuine or Forged signature to form a synthetic feature. According to the combination of *genuine signature* versus *genuine signature*, and *genuine signature* versus *forged signature*, the synthetic feature can be labeled into two types. For each experimenter, we randomly select 15 genuine signatures as reference samples denoted by $R_{i}$. *i* is the index of the experimenters. The remaining signature samples and its forged samples are denoted as $G_{i}$ and $F_{i}$, respectively. In the training stage, we first calculate a similarity vector $S_{G}^{i}$ between each pair of $G_{i}$ and $R_{i}$ with a label of *genuine*. Meanwhile, we also calculate similarity vectors $S_{F}^{i}$ between pairs of $G_{i}$ and $F_{i}$ with labels of *forged*. We train different classifiers with the above two types of samples as a training dataset to decide whether a new input signature is forged or genuine. The detailed model training process is shown in Figure 5. Unlike a traditional HSV system that requires a new user to enroll samples for model retraining, SilentSign can be conveniently used by a newly registered user with less retention, as our model determines thresholds to differentiate genuine or forged signatures in the verification process. We explore four different classifiers including logistic naive Bayes (NB) [41], linear regression (LR) [42], random forest (RF) [43], and support vector machine (SVM) [44] to search for an optimal one. We demonstrate in detail the comparison of different models in Section 6.2.4.

### 5.3. Signature Verification

An individual who wants to use SilentSign should first enroll his/her signatures in the database. In this way, their signatures are templates and labeled as *genuine*. Unlike a questioning individual who claims to specify user input as a querying signature, SilentSign will calculate the similarity vectors between querying signatures and stored signatures. Then the final classifier will distinguish whether the similarity vectors are in the genuine range.

## 6. Performance Evaluation

### 6.1. Acoustic Sensing

#### 6.1.1. Tracking Accuracy in 1-D

**Figure 6:**
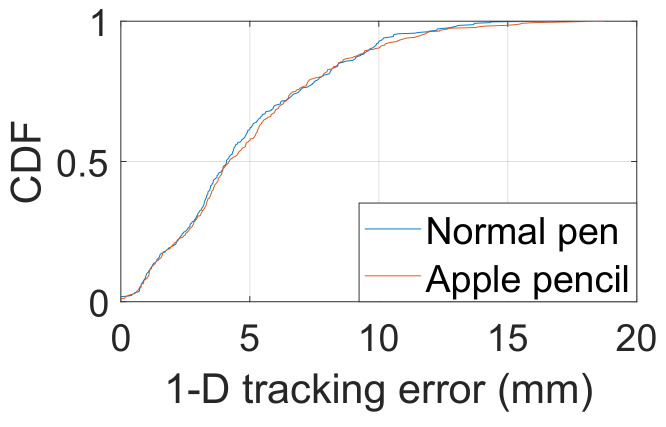
1-D tracking errors with a normal pen and the Apple pencil.

We evaluated the 1-D distance tracking in this section. We used a Samsung Galaxy Note 8 as the experiment device throughout all the evaluations unless otherwise specified. The system implementation used for our evaluation is based on Android Open Source Project (AOSP) v.8.1.0 (Oreo) Android is an open-source and Linux-based Operating System for mobile devices such as smartphones and tablet computers. Wide target audiences, low average price, and easy learning make Android the most suitable platform for the implementation of the SilentSign prototype. We use a millimeter ruler as a benchmark for ground truth and set 29.7 cm as the overall test distance, which is equal to the width of A4 paper. SilentSign continuously measures the distance between the smartphone and the moving pen while moving along with the scale of the ruler. The ground truth is the length of the line measured by the scale of the ruler. We also add the time-of-flight (TOF) of acoustic signals as a compensation factor to improve the precision of the measurement. Since the distance between the transmitter and receiver (i.e., the distance between the speaker and microphone of the Samsung Galaxy Note 8) is 0.7 cm, we can then calculate the TOF that the initial pulse spends on the LOS path. We also compared the distance estimation accuracy with normal ball-point pens (PILOT G1 Gel Ink Ball Point Pen, PILOT Juice Gel Ink Ball-Point Pen, etc.) and an Apple Pencil. Repetition of the above measurement 400 times allowed the cumulative distribution function (CDF) of distance estimation errors to be obtained, as shown in Figure 6. It is observed that SilentSign achieves average errors of 4.09 mm and 4.20 mm for the normal pen and the Apple Pencil, respectively. The errors in the 90 th percentile are 9.64 mm and 9.80 mm, similar to traditional digital signing devices.

#### 6.1.2. Tracking Range

**Figure 7:**
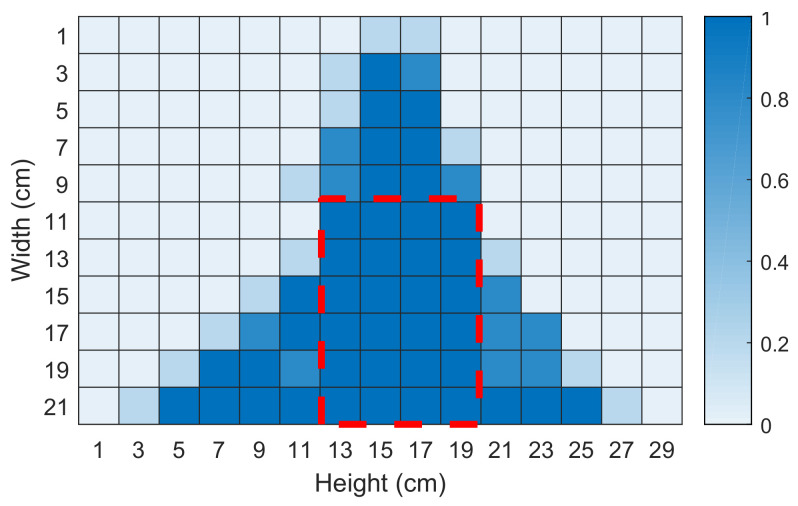
Acoustic sensing range.

We also carried out an experiment to evaluate the effective sensing range. First, we separate a piece of A4 paper (21 cm × 29 cm) into a grid of 609, each of which has an area of 1 cm × 1 cm. The smartphone will be placed above the central line of the landscape paper. After that, we draw circles on each grid. When the pen movement is detected within the sensing range, the path corresponding to its position will change and the initial value will appear in different impulse responses. For every block 609, we label it when there is an initial value in different impulse responses. We show the experimental results in Figure 7 where the dark cells mean a higher sensitivity. We can see that the optimal sensing area is the cells surrounded by a red dashed line. The distance between this square and the smartphone is 11 cm. Compared to a commercial product (e.g., Wacom STU-300 with a signing range of 2.5 × 9.9 cm^2^ [45]). Our system has a larger signing range, which is enough for signature verification. Therefore, we conduct the following evaluation experiments in this region.

### 6.2. Signature Verification

#### 6.2.1. Data Collection Setup

We conducted an experiment to obtain a set of genuine and forged signature data from several subjects. One of the goals of data collection is to collect skilled forged signatures. We then use an iPad Pro with an Apple pencil as a signing device, since we can turn on the screen record service while a reference signature is signed. Subjects acting as forgers can practice imitating genuine signatures by watching the recorded signing video until they are skilled. The screen displays the signing range as mentioned in Section 6.1.2 and the smartphone is placed above its signature position and turns on the SilentSign app to sense the movement of the pen tips. We chose a skillful forger by using the following process: each of their forgery attempts were compared to the genuine signature they were shown using DTW. A higher DTW score means that their signature trace is more similar. We then ranked the students according to these scores to select the appropriate number of skillfully forged signatures. Furthermore, the whole experiment is conducted in a rich-noise lab to ensure the system’s practicability. To protect personal privacy, we ensured that each participant’s signature data remained private and was only used in the experiment.

#### 6.2.2. Data Collection

We collected data from participants of 35 of different sex of different nationalities and ages from our university. The duration of the experiment is over one month to prove the time-invariance of the performance. The whole procedure includes the following two steps.

- *Step 1: collecting the Genuine Signatures.* We collect authentic signatures from 35 participants. Each participant was asked to write 20 signatures. The microphone tracks the vertical movement of the pen, and the trajectory is traced and logged using the screen recording function. In total, we have 700 signature samples from 35 participants;
- *Step 2: collecting the Forged Signatures.* We also created false signatures for the 35 participants. Each participant repeated one signature 20 times to create 20 samples for the signature. Afterward, 5 authentic signatures of 5 participants were arbitrarily chosen, which were to be replicated by all participants. Each signature was forged 4 times. At the beginning of the forging, participants first watched the video of a real signing. They practiced several times to maintain high confidence in their imitation. In total, 700 fake signatures were generated. Therefore, for one participant, she had 20 corresponding fake signatures, which were written by 5 different participants. The dataset statistic is shown in Table 1.

#### 6.2.3. Signature Verification Setup

We evaluated the reliability of SilentSign by making two comparisons. The first is between the authentic signatures and the random forged signatures. The second is between the authentic and the skilled (i.e., exercised) forger. Compared to the random forged signatures, we will learn the capacity of SilentSign to keep arbitrary signatures from acceptance. The skilled signatures were created with a certain level of training in the genuine signature of the claimed user [8]. Therefore, according to the above discussion, our evaluation of SilentSign is based on the verification of 3 cases, as shown below. Note that every participant (i.e., *u*) has 20 genuine signatures ($G_{i}$) and 20 forged ones (i.e., $F_{i}$).

- *Case 1:* skilled forgeries vs. genuine signatures (denoted as ‘SF’);
- *Case 2:* random forgeries vs. genuine signatures (denoted as ‘RF’);
- *Case 3:* genuine signatures vs. both forgeries (denoted as ‘ALL’).

We randomly chose real signatures from $G_{i}$ of *u* to apply to all verification cases. We arbitrarily used 15 forged ones in $F_{i}$. We also employed 15 participants’ (except *u*) real signatures as random signatures. We had 15 signatures for RF almost all the time. For ALL, the 15 signatures were arbitrarily adopted from the entire set of signatures.

Then, the similarity vectors (SF vs. genuine, RF vs. genuine, and RF vs. genuine) were computed and used as inputs for the classifier whose outputs are the verification decisions. We replicated each test case 50 times. Each replication was started from the random selection of the signatures. The purpose was to test the generality of SilentSign. We used the average values as the results. We also compared the performance of different classifiers, namely LR, NB, RF, and SVM.

Two criteria were utilized to evaluate our verification model. The first is the area under the curve (i.e., AUC). The second is the same error rate (i.e., EER). For AUC, the curve is the receiver operating characteristic curve (i.e., ROC). The larger the AUC, the better SilentSign performs. EER can be found on the ROC where the possibilities of identifying positive and negative results are the same. Low EER means higher reliability of SilentSign.

#### 6.2.4. Performance of Different Models

**Figure 8:**
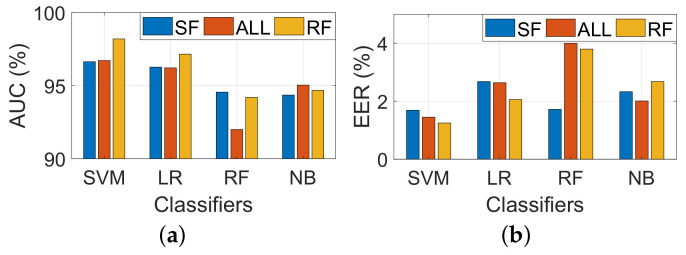
(**a**) AUC and (**b**) EER for different classifiers.

Figure 8 represents the performance of four different classifiers. It is observed that all classifiers achieve a higher AUC compared to 92% and an EER lower than 4%. This indicates significant differences in sign patterns between different people. It is also observed that SVM outperforms all other models with EER and AUC of 1.3% and 98.6%, respectively. This is because the feature used in our work is the similarity between the questions and the reference signatures. Genuine has a high probability of obtaining a higher similarity value compared to the forged one.

Specifically, we find that the RF task outperforms the other two tasks in SVM. ALL task has less AUC at 96.7% and EER at 1.5%. SF has the worst performance, which is 96.6% and 1.3%. The above is what we expected, which is that it is hard to distinguish skilled forgeries and genuine signatures because their distance variation is similar. Based on the above observation, we decided to use the SVM model as the final classification model in our scheme and evaluated another factor in the following section.

#### 6.2.5. Required Number of Reference Samples

**Figure 9:**
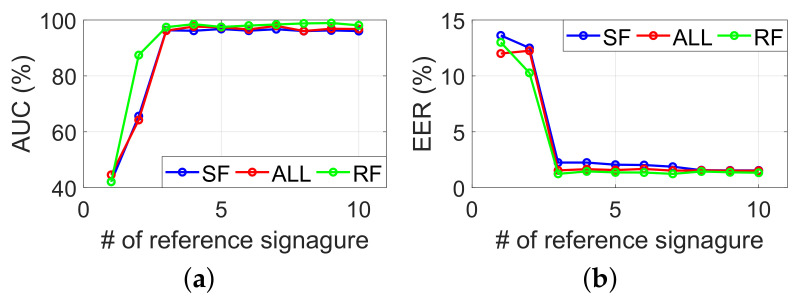
(**a**) AUC and (**b**) EER for various reference signatures.

As a trade-off between the security and user experiment, a required number of reference samples is very important. The number of reference samples in this experiment will gradually increase from 1 to 10, and the total number of subjects will be the same as in the previous section. We use the trained SVM model as mentioned in Section 6.2.3 to classify three tasks. Figure 9 represents the EER and AUC for a different number of reference samples. By increasing the number of reference samples from 1 to 10, the AUC improves from 42.1% to 97.4% and the EER reduces from 13.6% to 1.2%. The best performance is achieved at task RF when 9 signatures are used as a reference, with EER and AUC of 1.3% and 98.9%, respectively. According to our observation, even if three reference signatures bring about good performance, a more robust and secure system is obtained using more reference signatures.

#### 6.2.6. Required Number of Training Subjects

**Figure 10:**
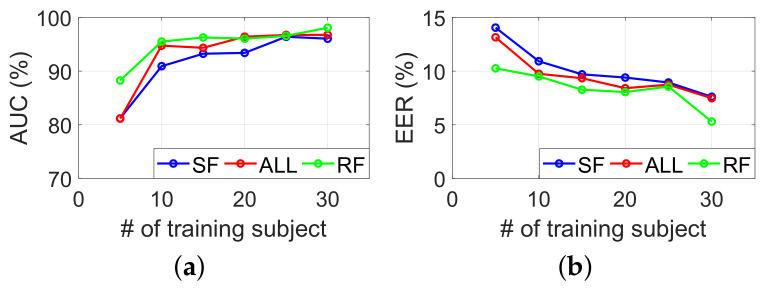
(**a**) AUC and (**b**) EER for different numbers of training subjects.

Here, we evaluated the impacts of the number of training subjects on system performance. For this purpose, we trained SilentSign with data from a varying number of randomly selected subjects. We then tested it with the remaining data. In each case, we repeated the training and testing process 25 times with a certain number of training subjects, to eliminate interference caused by abnormal samples. The average AUC and EER are represented in Figure 10. As expected, increasing the number of training subjects and scores is proportional to each other. We achieve the best performance in the RF task when the number of training subjects is 30, the AUC is 98%, and the EER is 5.3%.

#### 6.2.7. Impacts of Number of Forger Imitators in Training

**Figure 11:**
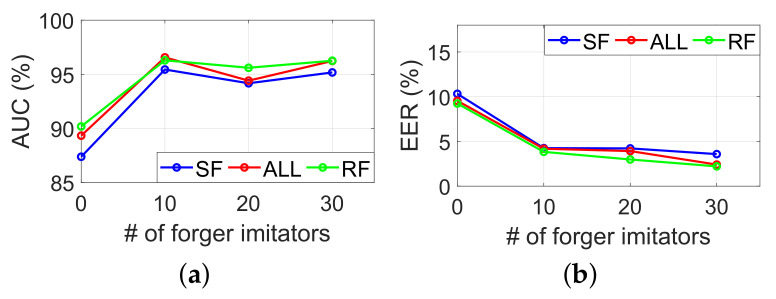
(**a**) AUC abd (**b**) EER under a various number of forger imitators included in the model training.

The function of the final SVM classifier in our system architecture actually acts as a boundary to delineate whether the similarity vector is in a certain range. The diversity of similar vectors of the training dataset is intended to make this bound more accurate. In the SVM training phase, we notice that it is difficult to collect the data of a real forger to calculate the similarity vector as training data. However, the forged signature in the training set can be collected from forger imitators who deliberately imitate our signature. Therefore, we conduct an experiment in order to figure out the impact of the number of forger imitators on performance. First, we randomly divide all the participants into three categories of legitimate users, real forgers, and forger imitators. Multiple SVM classifiers are trained with similar vectors from the legitimate user and forger imitators. The number of counterfeit imitators will gradually increase from 5 to 30. Then, we use similar vectors from legitimate users and real forgers as testing data. The result in Figure 11 shows that more forger imitators can improve the performance.

#### 6.2.8. Impact of Signature Complexity

More letters mean a more complex signature. It is difficult to forge a signature with more letters than one with fewer letters because it requires the forger to pay extra attention to the details of each letter. To understand the impact of signature complexity influence, we divide the data into three categories according to the number of letters. When a signature contained less than 4 letters, more than 10 letters, or in between, its complexity was defined as ‘*Simple*’, ‘*Complex*’, or ‘*Medium*’, respectively. We evaluate the AUC and EER when multiple subjects are randomly selected for each category. Table 2 shows that the higher complexity of the signature rapidly increases AUC and decreases EER at the same time. This meets our intuition that a more complex signature gives the forger more difficulty when forging, thus increasing security.

### 6.3. System Robustness

#### 6.3.1. Impact of the Smartphone Position

**Figure 12:**
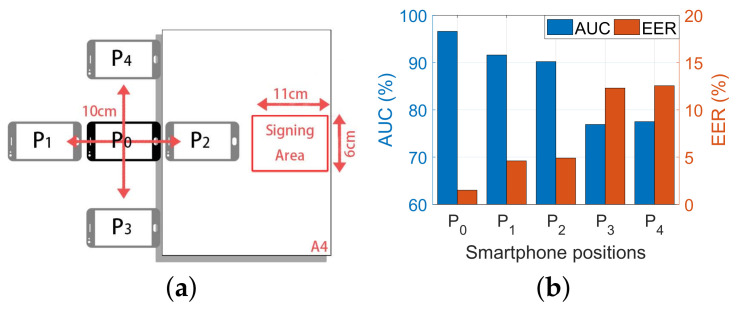
The performance when placing the smartphone at different positions. (**a**) The experimental setup of different position. (**b**) The AUC and EER in different positions.

Acoustic echoes are sensitive to the relative position between the position of the smartphone and the signing area. Therefore, we evaluated the performance under five different smartphone positions. Figure 12a shows the smartphone position($P_{0}$∼$P_{4}$), where the distance between each position is 10 cm. Each participant signs a signature on a piece of paper 10 times at each position on the smartphone. We used samples collected at position $P_{0}$ as a reference and samples at $P_{1}$∼$P_{4}$ as a query. Figure 12b shows the result. We notice that all the querying positions indeed degrade the performance; even if the distance change is the same, different moving directions will lead to a diversity of results. By moving the smartphone horizontally ($P_{1}$ and $P_{2}$), the performance is slightly decreased; in contrast, vertical movements ($P_{3}$ and $P_{4}$) result in a more apparent performance degradation. This is because vertical movement not only distorts the relative distance change more than horizontal movements but also changes the relative orientation of the phone and sign area. Thus, it increases the dissimilarity between the vertical position and the position $P_{0}$.

#### 6.3.2. Impacts of Smartphone Orientation

**Figure 13:**
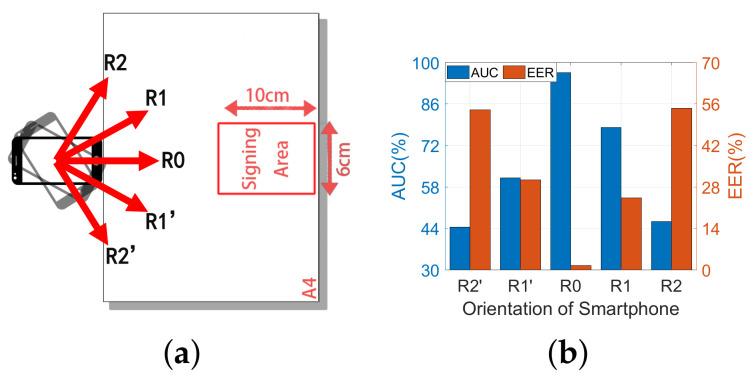
The performance when placing the smartphone at different orientations. (**a**) The experimental setup of different orientations. (**b**) The AUC and EER in different orientations.

In this part, we evaluate the impacts of a smartphone orientation by rotating it clockwise and anticlockwise by $30^{{}^{\circ}}$ with a step of $15^{{}^{\circ}}$ from the original position R0 as represented in Figure 13a. Two participants are requested to perform forged and genuine signatures, respectively, for 10 times at each orientation. As shown in Figure 13b, we verify the samples in R0 as reference signatures. The result shows that orientation R0 achieved the best performance, with AUC and EER of 96.7% and 1.5%, respectively. However, when the orientation deviates from R0, the system performance degrades considerably with AUC reducing to about 44% and EER increasing to about 54% in the worst case. Moreover, the greater the deviation, the worse the performance.

**Figure 14:**
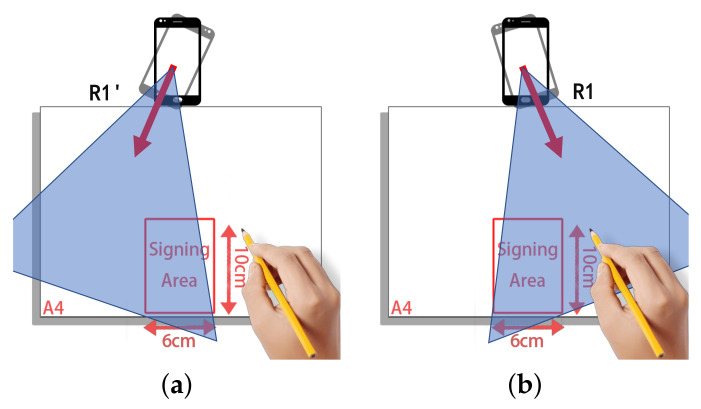
Experiment setting of the smartphone orientation. (**a**) The hand utilized to sign is out of the sensing range when the smartphone rotates 23 degrees clockwise. (**b**) The movement of the hand while signing can be captured by SilentSign since our hand is still within the sensing range.

We can also observe that the performance at R1 is better compared to $R1^{\prime }$. The reason is that when the smartphone is rotated clockwise by $15^{{}^{\circ}}$, the signing hand is out of the sensing range, as represented in Figure 14a, making SilentSign unable to effectively capture hand movements. On the contrary, signing movements can still be detected when the smartphone rotates to R2 as shown in Figure 14b. The results show that the orientation of the device is a critical factor that influences the performance of the system. Regarding the authentication performance, we recommend users to sign directly in front of the device.

#### 6.3.3. Impact of Noise

**Figure 15:**
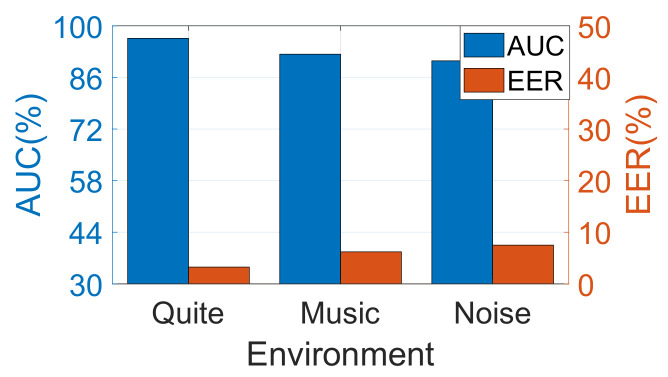
The AUC and EER under difference noise environments.

We also evaluated the performance of SilentSign under different noise levels. The data set was collected in the same indoor environment under three noise levels including ‘*Music* (40∼50 dB)’, ‘*Quite* (30∼40 dB)’, and ‘*Noise* (50∼60 dB)’. We use a sound level meter to guarantee that the ambient noise meets the requirements of the experimental setup. The model of the sound level meter is Smart Sensor™ AS824. Under the first condition, the participants conducted the experiments in a meeting room with only one air conditioner running. In the latter two cases, we played music and Gaussian white noise with corresponding power levels, respectively. We asked two females and two males to participate in the experiments with each writing down 20 signatures as a reference in the ‘Quiet’ environment and another 20 signatures in each of the remaining two environments. The average EER and AUC are represented in Figure 15. It is observed that the AUC of SilentSign under three conditions is greater than 90% with an EER varying from 3.2% (‘*Quiet*’) to 7.5% (‘*Noise*’). This indicates that SilentSign has a strong robustness to external noise. The underlying reason is that SilentSign uses high-frequency acoustic signals with fewer frequency overlaps with common noises.

#### 6.3.4. Robustness against Attacks

We evaluated the robustness of SilentSign against two different types of attacks, namely, random attack and imitation attack. In each case, we selected three new signatures (labeled $V_{1}$,$V_{2}$,$V_{3}$) as references from participants who were also recruited with data not used in model training. Since the complexity of the signature influences the difficulty of imitation, the reference signatures are carefully chosen by a complexity ranging from ‘*simple*’ to ‘*complex*’ based on the description in Section 6.2.8. In imitation attacks, a total number of 20 participants act as attackers, where each imitates a reference signature for 30 times. In random attacks, the attacker does not know about the conduction of a certain trajectory through a target user. Instead, we just let them know the letters within the victim’s signature before signing.

Unlike the screen recording in Section 6.2.2, an attacker imitates hand movements and signing trajectories by watching videos of a target’s signing process in imitation attacks. The reason is that attackers are more likely to capture detailed information (e.g., hand movements) of signing processes rather than only trajectories, to improve the imitation technique and attack effect. We evaluated the robustness against attacks with false acceptance rate (FAR) and summarized the results in Table 3. The average FAR in random and imitation attacks in three signatures is observed to be 1.6% and 4%, respectively. It is indicated that our system is more robust against random attacks compared to imitation attacks. The complexity of signatures is found to be beneficial to improve the robustness of the system against random or imitation attacks, as the FAR reduces from $V_{1}$ to $V_{3}$, according to the table. Moreover, the performance gap between the two types of attacks reduces from 4% to 0.2% along with significant complexity as well. This means that increasing the complexity of the signature is more effective in increasing the robustness against imitation attacks.

### 6.4. Computational Overhead

**Figure 16:**
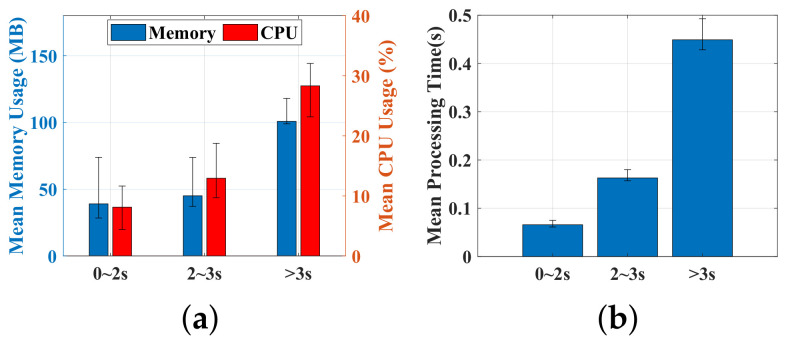
Computational overhead. (**a**) CPU and memory usage across different sample sizes. (**b**) Comparison of the processing times across various sample sizes.

In this section, we run the verification model on a server and measure its CPU usage and memory. The verification process was run 100 times. At each time, the test samples were randomly selected. According to the duration of signing processing, we separated the samples into three categories: 0∼2 s, 2∼3 s, and 3∼4 s. This is oriented by the intuition that a larger sample size leads to more processing time. Figure 16a compares the extreme value and means of each category. The average usage of memory and CPU for samples belonging to the above categories is observed to be (39 MB, 8.1%), (45.1 MB, 12.9%) and (100.8 MB, 20.3%), respectively. Furthermore, we also calculated the mean and extreme processing time as represented in Figure 16b. The samples in the first category yield the lowest latency of 0.065 s, which is much smaller compared to the samples in the latter categories by 0.098 s and 0.384 s, respectively. It is indicated that memory, processing time, and CPU usage are positively correlated with the duration of the signing process, which is in accordance with our intuition.

### 6.5. Comparison with Existing Work

In this section, we compare SilentSign with previous work including AirAuth [26], AirSign [15], Levy’s work [12], and ASSV [16] from aspects of utilized sensor, sensor size, model, signing area, and performance, and obtain the results as shown in the Table 4. Compared to them, our work is more related to ASSV [16]. However, ASSV [16] requires placing the phone parallel to the direction of the signature to grow linearly. There is a 9-millimeter length between the acoustic sensors and the writing area. Furthermore, the sign area is a 0.9 × 0.38 cm^2^ square, which is smaller than the commercial signature pad and our system. A narrow signing range will limit the user experience for some people signing their names on a large scale. Compared to the above work, Silentsign achieves 92.2% AUC by using the already existing speaker and microphone in most of today’s smart devices, which do not require hardware modifications. Furthermore, these two sensors will not squeeze any screen space. As for application scenarios, our experiment indicates that in the paper and pen signing scenario, to perform a signature verification, the finger can be used in our system as a tool for signing.

## 7. Discussion and Future Work

### 7.1. The Impacts of Forged Ground Truth Deficiency

The challenge of acquiring real forged ground truth in a practical application scene may affect the evaluation results. We consider using data augmentation techniques such as the generative adversarial network (GAN) [46], which is more powerful in generating approximate forged signatures. We hope that this scheme with a larger forged signatures dataset will give us a more robust model and that SilentSign will also be received in a real-life scenario.

### 7.2. Varying Smartphone

A potential limitation of our work is the position of the speaker and the microphone on smartphones. We implement our data collection application with a specific volume of 10. In cases where the speaker and microphone are located on different sides of the smartphone, the microphone may receive a weaker echo, resulting in a lower IR estimation performance. Further, changes in the distance between the speaker and the microphone affect the compensation factor, leading to tracking errors.

## 8. Conclusions

In this study, we present SilentSign, an acoustic-sensing-based HSV approach running on portable devices. Compared to the traditional HSV scheme, our scheme possesses lower hardware requirements. SilentSign offers several advantages, such as security, accessibility, and support for signing on paper materials. Our scheme merely relies on ubiquitous acoustic sensors and is a purely software-based scheme. The acquired results indicate that our well-designed SilentSign system achieves 98.2% AUC and 1.25% EER. Although it still has restrictions in robustness and practicability, we believe it to be an auspicious technology deserving of further research.

## Figures and Tables

**Table 1:** Dataset statistic.

Female	Male	Age 18∼24	Age 24∼28	Age 28∼32	# Genuine	# Forge
18	17	11	12	7	700	700

**Table 2:** AUC and EER for different signature complexities.

	AUC (%)			EER (%)		
	SF	ALL	RF	SF	ALL	RF
Simple	83.1	85.9	85.5	18.9	16.8	16.7
Medium	88.8	91.9	94.4	11.5	6.9	3.1
Complex	93.8	92.4	96.1	3.4	4.2	3.8

**Table 3:** Results of random and imitation attacks.

Victim	Random	Imitation
$V_{1}$ (simple)	2.7	6.7
$V_{2}$ (medium)	1.7	4.6
$V_{3}$ (complex)	0.6	0.8
Average	1.6	4

**Table 4:** Comparison of various signature verification systems.

	Sensor	Model	Sensor Space	Signing Range	Accuracy
[15]	acoustic and motion sensors	MD-DTW+KNN	Small	$25\times 25\;{cm}^{2}$	97.1% F1-score
[26]	depth camera	DTW	Large	base on camera focal length	96.6% EER
[12]	motion sensors	SVM	None	anyplace on the paper	98.5% AUC
[16]	acoustic and microphone	CNN	None	$0.9\times 0.38\;{cm}^{2}$	98.7% AUC
**Our work**	**microphone and speaker**	**SVM**	**None**	$7\times 11\;{\mathrm{cm}}^{2}$	**98.2% AUC**

## Data Availability

Not applicable.
